# Remarks on the Enucleation of Uterine Fibroids

**Published:** 1876-10

**Authors:** T. Gaillard Ttomas


### Remarks upon the Enucleation of Uterine Fibroids, with Illustrative Cases.

By T. Gaillard Ttomas, M. D.

One of the most valuable contributions made by America to gynæcological surgery, emanated from W. L. Atlee, in reference to the management of sessile sub-mucous and intrestitial uterine fibroids. In the year 1853 he presented to the American Medical Association an essay entitled "The Surgical Treatment of Certain Fibrous Tumors of the Uterus heretofore considered beyond the resources of Art." This essay received the prize of the association, and to-day stands as the pioneer article in the surgical literature of these grave and often irremediable cases.

Both in this country and in Europe the lead of this bold surgeon has been followed, and the method which he advocated a quarter of a century ago, and which slowly battled with a pretty decided opposition has come to be recognized as a legitimate surgical resource.

If I may be allowed to epitomize the views of Atlee, as published in 1853, I would do so in these three propositions.

*First*—If a non-pediculated tumor cannot, from the nature of its attachment and envelopes be expelled or drawn by mechanical means through a dilated os uteri, it is advisable to make by the knife a means of escape for it into the uterine cavity, through its capsule or enveloping tissues.

*Second*—If the tumor, thus offered an outlet, cannot be removed, it should be forced into and out of the uterine cavity by persistent use of ergot and cutting the cervix.

*Third*—The tumor, once coming within reach, it should as soon as practicable be enucleated and removed by the surgeon.

That this method of treating such cases is attended by the great dangers of septicæmia, peritonitis, hemorrhage, and exhaustion, is not to be denied. But it must be borne in mind that while heroic interference is environed by risks, a Fabian course of non-interference and inactivity is by no means a safe one. The growing tumor creates exhausting hemorrhage, dangerous mental depression and anxiety, and interference with the functions of nutrition and excretion, which slowly drag the patient down to death. Interference should not be practiced unless impending danger urges a resort to it. Cases selected by this rule commonly end in recovery, while non interference commonly results in death.

Although many surgeons here and abroad have, as I have already said, followed the lead of Atlee in this matter, it is still of importauce that a fair report of cases thus treated should be made in order that those believing may be strengthened, and that those doubting may be convinced. In this spirit I report the following cases.

CASE 1.—Large Fibroid expelled through opening made in its capsule.

Mrs. C., residing at Red Hook, N. Y., æt. forty years, been married thirteen years, the mother of one child eight years of age, called upon me by advice of Dr. Bates, of Rhinebeck, and gave me the following history of her case. Four years ago her menstrual periods had ceased for six months, and she began to think that the menopause or pregnancy had occurred, when suddenly they reappeared. At the same time she was disturbed by noticing that her abdomen was enlarging.

From this time the menstrual discharge became profuse, the health depreciated and the strength greatly diminished. The abdominal enlargement steadily increased meanwhile, and at the time that she applied to me, my note book records it as being "as large as in utero-gestation between the seventh and eighth months."

Upon her visit to me, on the 9th of June, 1875, I found Mrs. C. very pale, thin, weak and bloodless. The appetite was poor, digestion feeble, pulse rather weak and rapid, and the patient's mind much depressed about her condition.

Physical examination revealed the upper portion of the cervical canal expanded as at the commencement of labor, the walls of the cervix thin, and a tumor filling the cavity above, and firmly attached to the walls of the cervix, except on one side, the posterior. The uterine sound on this side passed up about five inches, but everywhere else the growth was attached all the way down to the lowest portion of the cervical canal. The tumor which presented was rather soft, aud I suspected that it might be fibrocystic instead of purely fibrous. The uterus had, from the history of the case, evidently made determined efforts to expel it; but on account of the resisting envelope, had entirely failed in doing more than dilating the os externum.

The patient being unwilling to remain in town, I decided. *First*, To pass a large aspirator needle into the mass, to ascertain if it contained spaces filled with fluid. *Second*, If it did not do so, to make an opening into the capsule which would constitute an artificial os for the mass. *Third*, To give ergot steadily to excite expulsive efforts on the part of the uterus to force out the growth.

Accordingly on the 10th of June, with the assistance of Drs. H. F. Walker, and S. B. Jones, Jr., this course was inaugurated at the patient's hotel, and on the next day she returned to Red Hook without inconvenience.

I did not hear again from her until a fortnight afterwards, when she wrote that ever since she had returned home, she had suffered from uterine pains of intermittent character, and slightly bloody flow of a disagreeable odor.

From her attending physician, I subsequently ascertained the progress of the case. The pains referred to steadily forced down the tumor through the opening made in the capsule. It presented exactly as a child's head would have done, and after between two and three weeks of a process closely resembling labor, it distended the perinæum and by a very firm traction on his part, was delivered. During this time, a most offensive odor was given forth by the mass, and the patient suffered from a certain degree of septicæmia. Unfortunately the tumor which was large, decomposed, and almost diffluent, was not weighed.

Subsequent to this, Mrs. C. entirely recovered, and now, one year afterwards, is, I believe, in good health.

I neglected to say that the attempt at aspiration yielded no fluid whatever. It is probable, however, that the acupuncture resulted in the partial death of the badly organized mass and aided materially in exciting expulsion.

The second case which I shall record was seen in consultation, and as a report of it, made by the attending physician, has not been published, I avail myself of his kind permission to employ his manuscript here.

Case 2.—Sub-mucous Uterine Fibroid. Uterine contractions excited by ergot. Tumor enucleated by Prof. Thomas. Reported to the District Medical Society of Bergen County, New Jersey, by Chas. Hasbrouck, M. D.

Mrs. A., aged forty, first menstruated at fifteen, married when twenty years old; has had four children, the eldest nineteen, the youngest between ten and eleven years old; never had an abortion or miscarriage, and until recently never had any serious illness. Previous to the birth of her last child, Mrs. A. always enjoyed robust health. In the winter of 1863-4, a few weeks after the birth of her last child, and before she had recovered fully from the puerperal condition, her husband had an attack of typhoid fever and was very ill for several weeks, and Mrs. A. became very much worn down from protracted watching, fatigue and anxiety. Soon after this, she began to suffer from sleeplessness and general nervous irritation. Her appetite remained good, and her nutrition seemed perfect, with rather a tendency to the accumulation of fat; her menstrual functions were regular and without pain or any abnormal symptom; she never had leucorrhea, backache, nor any other symptom of uterine disease. But at the same time she continued to suffer from general nervousness, sleeplessness, and neuralgic pains in the back of her head, shoulders and chest, down to the waist. Her spine was more or less tender, and she sometimes had a nervous, dry cough, and sometimes vomiting, and almost constantly suffered more or less from the protean forms of hysterical disorders. During all this time her menstrual functions were naturally performed, and on careful examination, by touch and by the speculum, no uterine disease could be discovered.

In 1868-9, Mrs. A. began to menstruate rather scantily, although regularly as to time, and became more and more nervous, requiring almost the daily use of chloral-hydrate and bromide of potassium to relieve her nervous disorders and wakefulness.

In 1870-1, she began to menstruate more freely. The catamenia continued to recur at the regular time, and continued to be free from any kind of suffering, but they gradually became more and more profuse, until finally it amounted to actual menorrhagia and began to tell upon her strength. This condition continued for several months before my attention was called to the fact; and increased debility, and aggravation of her sleeplessness and general hysterical distresses were the results.

Finally, during the past summer, 1873, my patient called my attention to the fact, that notwithstanding her increasing debility, and very appreciable emaciation, there was a noticeable increase in the size of her abdomen; and on examination, I discovered a distinct circumscribed tumor in the hypogastrium, symetrical, or nearly so, in form, and about the size of the uterus in the fifth month of pregnancy. The tumor was evidently uterine.

By the persistent use of astringents and perfect rest in the horizontal position during the menorrhagic flow, and of tonics, quinia and iron, during the intervals, the amount of the hemorrhage was very materially lessened, and the general health of the patient improved. But the hypogastric tumor remained, and perhaps increased very slightly in size, disturbing the patient's mind and interfering with her general comfort.

November, 1873, I got Prof. T. G. Thomas, of New York, to see Mrs. A., and after a careful examination he expressed the opinion that the tumor was undoubtedly uterine, and most probably a uterine fibroid. But in view of the fact that it might *possibly, but not probably*, be one of the rare cases in which pregnancy existed with regular menstruation, he declined to risk the danger of resorting to the use of the probe or uterine sound, which was necessary to perfect the diagnosis, until about six weeks or two months had elapsed. by which time the existence or non-existence of pregnancy would be developed with entire certainty.

February 20, 1874, Prof. Thomas again saw the patient, the uterine tumor had increased slightly in size, but the non-existence of pregnancy beiug sufficiently evident, he did not hesitate to use the sound, and found the uterine cavity to measure about five inches. He diagnosticated the presence of fibroid tumor of the uterus of the sub-mucous variety; and advised the persistent use of ergot, in the hope of starving out the tumor, or at least, retarding its farther developement by diminishing its blood supply; and in the farther hope that the uterus might be induced to take on expulsive action and expel the morbid growth. In accordance with this advice, I gave Squibb's solid extract of ergot in four grain doses three times a day, beginning February 21, 1874.

March 2, Mrs. A., began to suffer from severe pains in the iliac and hypogastric regions. These pains were constant but aggravated in paroxysms. They were evidently uterine, and no doubt the result of the ergot. These pains contined with scarcely an interval of ease, and finally became so severe and exhausting that I was obliged not only to discontinue the ergot, but to resort to hypodermic injections of morphia to relieve the terrible suffering. Even after the discontinuance of the ergot, the pains continued to recur daily between twelve o'clock M. and one o'clock P. M., generally requiring a dose or two of morphia to procure a night of rest.

In the meantime the cervix uteri gradually softened down and the os uteri became patulous, so as to admit the first phalanx of my finger, when I could reach the lower portion of the tumor. The pains still continued to recur daily. The os uteri became more and more soft and dilated, until it reached the size of a dollar, the lower portion of the tumor apparently becoming somewhat detached and gangrenous, filling up the os, and emitting a terribly offensive odor. I attempted with a strong polypus forceps to remove the offensive presenting mass, but could only tear away a part of the putrid portion, while as far as I could reach with my finger, I could feel the tumor firmly imbedded, apparently in the posterior and lateral walls of the uterus.

The constant suffering of my patient from the recurring pains, loss of sleep, etc., greatly exhausted her. Besides, her pulse became frequent and irritable, and her skin was almost constantly bathed in a profuse perspiration, while toward morning she sweated so profusely as to drench her clothing and the bed clothes. The discharge from the gangrenous mass became more and more offensive and profuse, and it became evident that my patient would die from septicæmia and exhaustion if the efforts of the uterus to rid itself of the offending tumor were not aided by the judicious application of art. Under these circumstances, I telegraphed to Prof. Thomas, to visit the patient and adopt such farther measures as he might deem necessary and expedient.

March 18, 4 o'clock, P. M., Prof. Thomas visited the patient with me; and in view of her weakened condition, the size of the tumor, its extensive attachments, and the great danger to the patient from any farther delay, he advised the immediate removal of the tumor, if possible, by enucleation.

Accordingly Mrs. A., was placed fully under the influence of ether, and removed to a table in a strong light. Sims' speculum was introduced, when the tumor could be seen filling up the partially dilated os. Dr. Thomas seized it with strong forceps, but it was so putrid as to tear on making traction. After removing as much as possible in this way, the doctor succeeded in passing the loop of an écraseur around a part of the remaining undecayed portion of the tumor and removed another large piece, the wire of the écraseur breaking during the process. Having thus cleared the os and cervix of a considerable portion of the tumor, he next, partly by the use of an enucleator, and partly by a process of clawing, succeeded in entirely removing the mass, the whole process occupying upwards of an hour.

Mrs. A., was then carried to bed after the uterus had been freely washed out with carbolized water, and the effects of the ether allowed to pass off. She vomited several times, pulse frequent and feeble. Brandy and water were given *ad libitum* and a hypodermic injection of morphia gr. ss. was administered.

March 19, A. M.—Has passed a sleepless night notwithstanding the free use of brandy and morphia. Pulse, ninety-six : temperature, ninety-nine. Loathes food; perspires profusely; feels terribly sore.

P. M.—Pulse ninety-six ; temperature ninety-nine and a half. Treatment—Quinine gr. iij ter in die: beef-tea and milk; morphia hypodermically and by mouth in sufficient doses to procure rest. Three grains have been taken during the day.

March 20, A. M.—Pulse ninety; temperature one hundred; discharge slight and not so offensive as before operation ; continued treatment. The uterus is washed out twice a day with carbolized water, by means of elastic catheter introduced quite up to fundus.

P. M.—Pulse, eighty-five; temperature, ninety-nine and a half ; the discharge becoming more free and offensive, but not so much so as before operation. Rests tolerably; still sweats profusely in the morning.

Without giving a detailed statement of the farther progress of the case, I will simply state that from this time Mrs. A., progressed favorably. Her profuse sweats gradually ceased; she soon began to crave food; the uterus soon subsided so as scarcely to be felt above the pubes. A few shreds of putrid matter were washed away by the injections, but the discharge soon ceased entirely, and in a short time the patient was sitting up, still feeble but apparently well, in much better health, at all events, than for several years past. The tumor, as nearly as could be estimated from the pieces, was about as large as a small cocoanut.—*Archives Clinical Surgery.*

